# Enhancing the inhibition and adsorption performance of SABIC iron corrosion in sulfuric acid by expired vitamins. Experimental and computational approach

**DOI:** 10.1039/d1ra01010g

**Published:** 2021-05-11

**Authors:** M. Abdallah, K. A. Soliman, Arej S. Al-Gorair, A. Al Bahir, Jabir H. Al-Fahemi, M. S. Motawea, Salih S. Al-Juaid

**Affiliations:** Chemistry Department, Faculty of Applied Science, Umm Al-Qura University Makkah Saudi Arabia maabdelsaid@uqu.edu.sa metwally555@yahoo.com; Chemistry Department, Faculty of Science, Benha University Benha Egypt; Chemistry Department, College of Science, Princess Nourah Bint Abdulrahman University Riyadh Saudi Arabia; Chemistry Department, Faculty of Sciences, King Khalid University Abha Saudi Arabia; Chemistry Department, Faculty of Science, Tabuk University Tabuk Saudi Arabia; Chemistry Department, Faculty of Science, King Abdulaziz University Jeddah Saudi Arabia

## Abstract

The inhibition potency of expired thiamine or vitamin B1 (VB1) and riboflavin or vitamin B2 (VB2) against SABIC iron corrosion in 0.5 M H_2_SO_4_ solutions was investigated using chemical and electrochemical techniques. Theoretical studies such as DFT and MC simulations were performed on both VB1 and VB2 inhibitors to obtain information related to the experimental results. It has been found that the inhibition efficacy assigned from all measurements used increases with increasing concentration of the two expired vitamins and reduces at elevated temperatures. It reached 91.14% and 92.40% at 250 ppm of VB1 and VB2, respectively. The inhibition was explicated by the adsorption of the complex formed between expired vitamins and ferrous ions on the SABIC iron surface. The adsorption was found to obey the Langmuir isotherm model. Galvanostatic polarization demonstrated that the two expired vitamins act as an inhibitor of the mixed type. These expired vitamins have proven effective in inhibiting the pitting corrosion induced by the presence of Cl^−^ ions. The pitting potential is transferred to the positive values showing resistance to pitting damage. The theoretical parameter values are consistent with experimental results.

## Abstract 2

The inhibition potency of expired thiamine or vitamin B1 (VB1) and riboflavin or vitamin B2 (VB2) against SABIC iron corrosion in 0.5 M H_2_SO_4_ solutions was investigated using chemical and electrochemical techniques.

## Graphical abstract

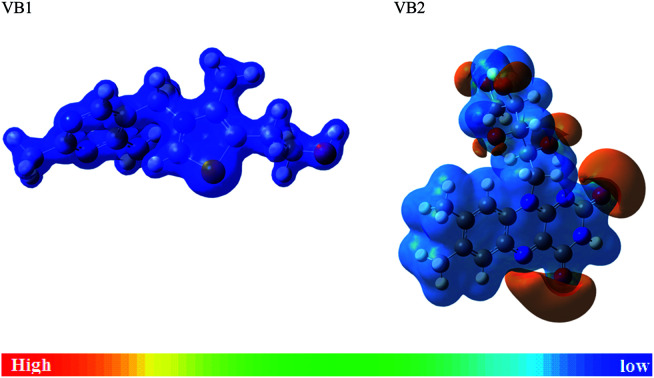

## 1. Introduction

Saudi basic industries corporation (SABIC) is considered one of the leading companies in the production of iron in the Kingdom of Saudi Arabia and the Arab Gulf region, and it is considered one of the largest companies producing iron and steel in the world. SABIC iron has multiple applications in many industries such as construction, building, automobile, agricultural machinery production, and many other industries. It is known that sulfuric acid is used in the pickling and cleaning of iron by removing grease and layers deposited on the surface, and unfortunately, this causes the corrosion of iron, which causes large losses to the national economy. For this reason, scientists have tended to solve this major problem in different ways, and one of these ways is the use of corrosion inhibitors.^1–5^

Several synthetic organic molecules^6–20^ were utilized to inhibit the corrosion of iron and steel in strong acidic media. Usually, these molecules containing heteroatoms and multiple bonds. The inhibiting performance of these molecules owing to its strong adsorption on the surface of the metal. The vigor of the adsorption depends on the chemical formula of the organic compound used as a corrosion inhibitor, the kind of the metal or alloy used, the temperature, concentration of hydrogen ions, the presence of electron-donating or repelling groups, the possibility to form complex.^21,22^

Most of the organic compounds are often given high inhibition efficiency and reduce the rate of corrosion, but most of these compounds are poisonous and their use is harmful to human health and the environment. Therefore, researchers try to use safe and harmless compounds, such as expired drugs. Currently, expired drugs have been used as corrosion inhibitors instead of fresh drugs.^23–25^ These drugs are not economically feasible, and according to the WHO report, we preserve at least 90% of their chemical composition even after the expiration date, but they are not used as a treatment for patients for medical purposes. But it can be used as a corrosion inhibitor which leads to solving environmental problems and has an economic value as it reduces the disposal costs of expired medicines.

The inhibiting strength of fresh or expired drugs depends on its chemical structure and the occurrence of some energetic centers that facilitate the adsorption of these drugs on the metal surface.^26–30^ Consistent with experimental work, computational chemistry based on density functional theory (DFT) and Monte Carlo methods became important tools for predicting the efficiency and reactivity of the chemical compound.^31–33^ Determination of the electronic characteristics of the compounds and the interaction between the compound and the metal surface have a relationship with the efficacy of the inhibitor.

The main objective of the current manuscript is to contribute to solving the problems resulting from the phenomenon of corrosion of SABIC iron in aqueous solutions by using expired pharmaceutical drugs namely, vitamin B1 (VB1) and vitamin B2 (VB2) that do not benefit and harm the surrounding environment and to link the experimental inhibition results with calculated by the computer. Instead of getting rid of them, they are used as corrosion inhibitors. These expired drugs are inexpensive, environmentally friendly and economically feasible. Chemical and electrochemical and theoretical studies were applied to confirm the inhibition efficiency of the studied drugs. The effect of high temperatures was also studied, and the thermodynamic activation coefficients were calculated and interpreted.

## 2. Experimental techniques

### 2.1. Electrodes and electrolytes

All experiments were conducted on SABIC iron with a purity of 99.99%. A.R. grade H_2_SO_4_ was used for preparing the corrosive solution (0.5 mg l^−1^ H_2_SO_4_). The concentration range of the drug inhibitors used was 50–250 mg l^−1^ by dissolved in binary distilled H_2_O.

### 2.2. Chemical and electrochemical measurements

For weight reduction (WR) experiments a SABIC iron coupons with dimension (1.5 cm × 3.0 cm × 0.1 cm) were utilized. Prior to any experiment, the coupons were polished with grades of sandpapers (ranging from 200 to 1000), washed with binary distilled water and finally with acetone.

After careful weighing, SABIC iron coupons were dipped in the tested solutions. After 6 hours the SABIC iron coupons are ejected, washed, dried and weighed carefully. After that, the experiment is reduplicate three times and the average is taken.

The corrosion rate was determined according to the next relation:^34^

1 *R*_corr._ = Δ*W*/*At*

where, Δ*W* (mg) is the difference in the weight reduction, *A* is the surface area and *t* is the inundation time.

For galvanostatic polarization (GP), potentiodynamic anodic polarization (PAP) and electrochemical impedance spectroscopy (EIS) measurements are performed in electrolytic cell it included three electrodes, which is a SABIC iron electrode with a surface area of 0.42 cm^2^, platinum counter electrode and a saturated calomel electrode (SCE). Before starting measurements. The SABIC iron electrode remains in the tested solution until the electrode potential remains constant (about 30 minutes). All measurements were performed at 303 ± 1 K.

Measurements were carried out with the Gamry (PCI 300/4) Potentiostat/Galvanostat/ZRA instrument. This involves a Gamry frame system based on the ESA 400. Gamry applications include DC105 for GP and PDAP measurements and EIS300 for EIS analysis coupled with a computer to collect data. GP and PDAP measurements were performed at 5 mV s^−1^ and 1 mV s^−1^, respectively. EIS measurements were accomplished in the frequency range of 100 kHz to 10 mHz with amplitude of 5 mV peak-to-peak amplitude using AC signals at open circuit potential.

### 2.3. Calculation of inhibition efficacy

The percentage inhibition efficacy (% *P*) and surface coverage (*θ*) of the suggested expired vitamins from WR, GP, and EIS measurements was determined using the subsequent equations:^35^2
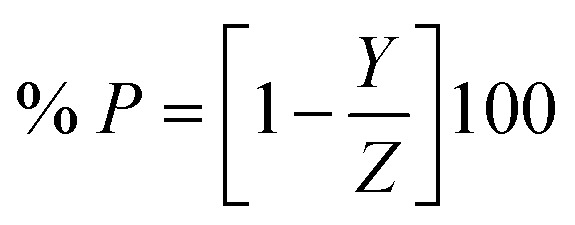
3
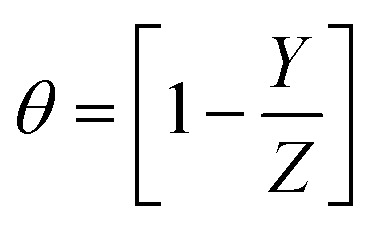
where *Y* and *Z* denote weight reduction losses or *I*_*c*orr._ in the case of WR and GA tests in the presence of VB1 and VB2 and in free 0.5 M H_2_SO_4_ solutions, respectively. In the case of EIS measurements the opposite is true, *Y* and *Z* symbolize the charge transfer resistances (*R*_ct_) in free 0.5 M H_2_SO_4_ solution and in the occurrence of the additives (VB1 and VB2) solutions, respectively.

### 2.4. Additives

The expired drugs used as inhibitor for the corrosion of SABIC iron are namely thiamine or vitamin B1 (VB1) and riboflavin or vitamin B2 (VB2) have been investigated purchased from Egyptian International Pharmaceutical Industries company (EIPICO). Expired vitamins are used in this study after 30 day of their expiration date. The names and chemical structures of expired VB1 and VB2 are given in Table 1.

**Table 1:** The names and chemical structures of expired VB1 and VB2

Name	3D shape	Chemical structure	Chemical formula and molar mass
Thiamine (VB1)	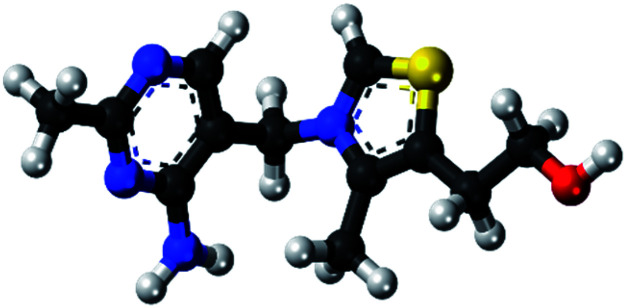	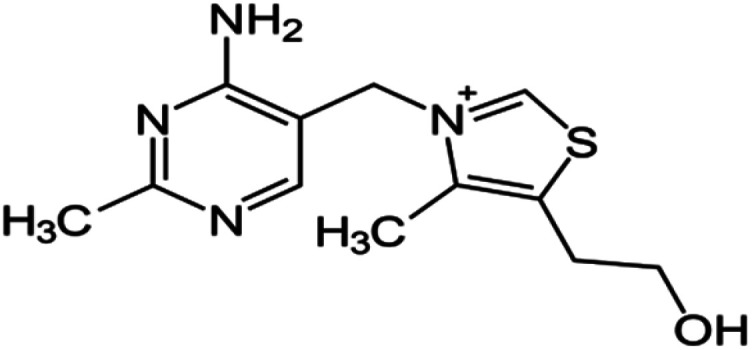	C_12_H_17_N_4_OS^+^, 265.35 g mol^−1^
Riboflavin (VB2)	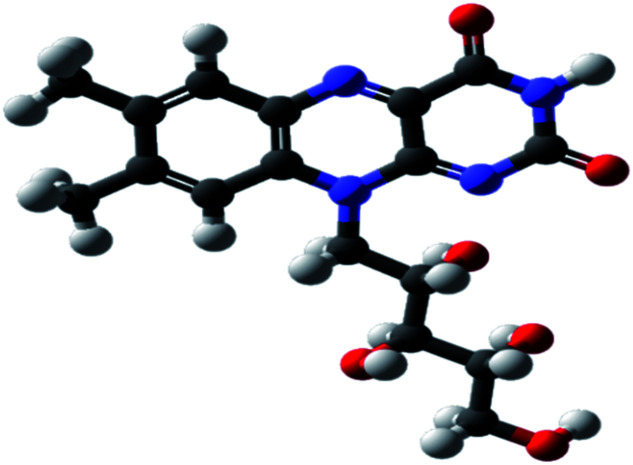	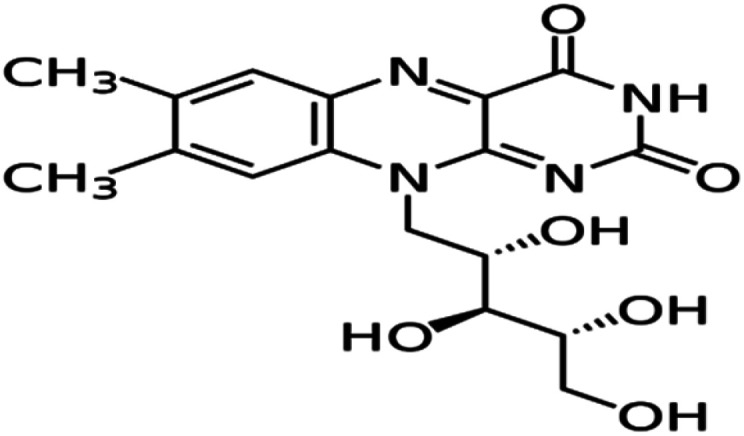	C_17_H_20_N_4_O_6_, 376.369 g mol^−1^

### 2.5. Computational details

The molecular geometry of VB1 and VB2 were firstly optimized based on DFT. In the present work, all the DFT calculations are derived from the use of the G09 program.^36^ For optimization, the exchange-correlation has been investigated by hybrid B3LYP functional^37^ and 6-31g(d,p) basis set. The optimization process was carried in the gas and aqueous phases without symmetry constraint. CPCM model was used for the solvent effect.^38^

Some important quantum descriptors were determined that are correlated with experimental inhibition efficiency such as energy of the highest occupied and the lowest unoccupied molecular orbital (HOMO & LUMO), the energy gap (Δ*E*), dipole moment (*μ*), global hardness (*η*), softness (*σ*) and the fraction of electron transferred (Δ*N*).

The global hardness and the fraction of electron transferred were computed by the next equations:4
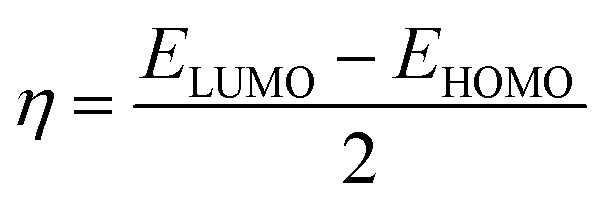
5
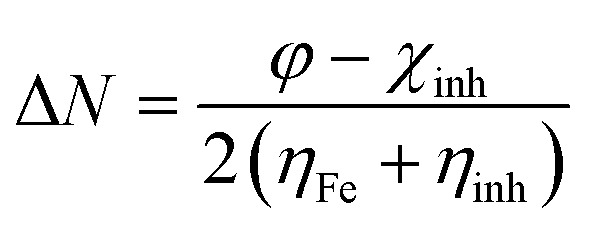
where *φ* is the work function of Fe and equal 4.82 eV for Fe(110),^39^*χ*_inh_ is the electronegativity of the inhibitor, *η*_Fe_ is equal to zero, and *η*_inh_ is the hardness of the inhibitor.

Also, we investigated the molecular electrostatic potential maps (MEP) and Fukui functions or indices. Fukui indices were determined using Mulliken charges of atoms. Fukui indices give knowledge about the local reactivity of the inhibitor. The electrophilic and nucleophilic attacks were investigated by Fukui indices *f*^−^ and *f*^+^ which calculated as follow:

6 *f*^+^ = *q*(*N* + 1) − *q*(*N*), for nucleophilic attack

7 *f*^−^ = *q*(N) − *q*(*N* − 1), for electrophilic attack

where *q*(*N* + 1), *q*(*N*), and *q*(*N* − 1) are the charges of anionic, neutral and cationic species respectively.

### 2.6. Monte Carlo simulations (MS)

MC carried by using the simulated annealing method to identify the adsorption of expired VB1 and VB2 on the SABIC iron surface. The Fe(110) was designed by cleaves the bulk iron with planes (110) then enlarge the surface to make a supercell of (6 × 6) with a vacuum 20 Å above the surface. The 3D simulation box consists of six layers of iron to place the optimized VB1 and VB2 inhibitors near the Fe(110) surface. The COMPASS force field was utilized to find the lowest adsorption energies of expired VB1 and VB2 on the Fe(110) surface.

## 3. Results and discussion

### 3.1. GP technique

**Fig. 1:**
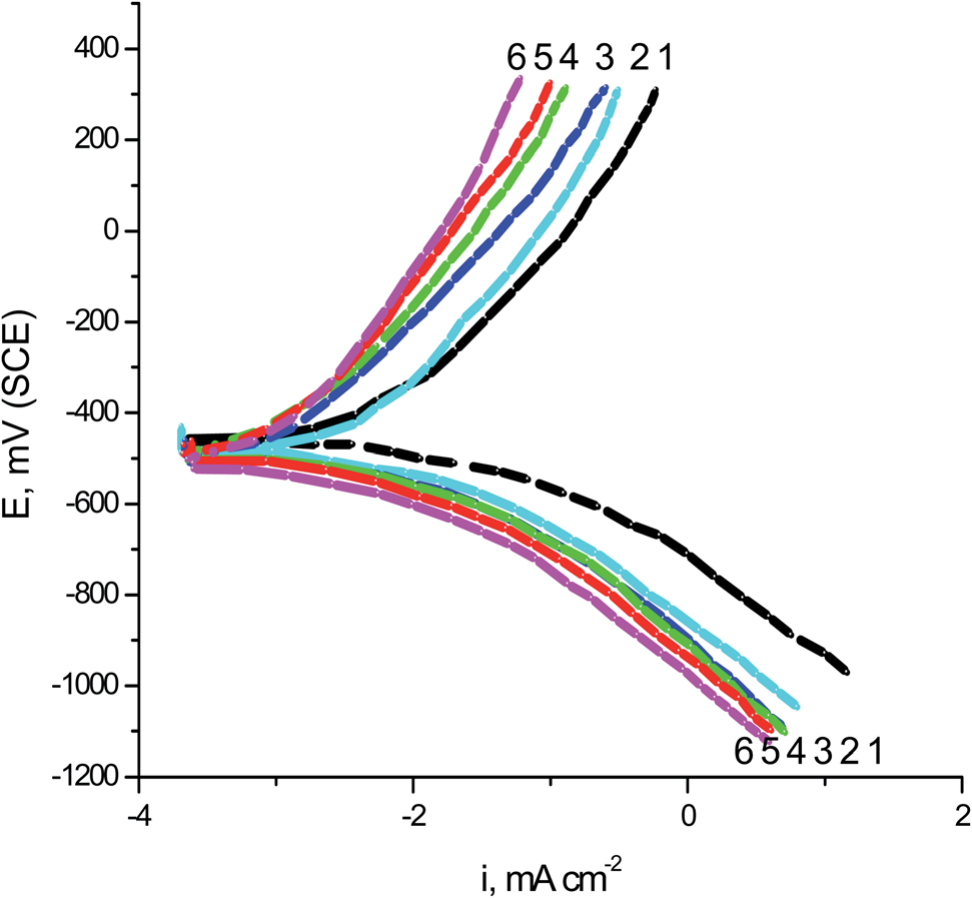
GP curves of SABIC iron blank 0.5 M H_2_SO_4_ solution and contains diverse concentrations of VB2. (1) 0.00 mg l^−1^, (2) 50 mg l^−1^, (3) 100 mg l^−1^, (4) 150 mg l^−1^, (5) 200 mg l^−1^ and (6) 250 mg l^−1^.

The GP curves of SABIC iron in the blank 0.5 M H_2_SO_4_ solution and contains diverse concentrations of VB2 was represented in the Fig. 1. Analogous curves were obtained for the other expired VB1 but not visible. Cleary the both the anodic reaction of SABIC iron and cathodic H_2_ reduction reaction are retarded when the two tested expired VB1 & VB2 are added to the 0.5 M H_2_SO_4_. This inhibition is more effective with increasing expired drugs concentration. The anodic and cathodic Tafel lines were moved to more positive and negative potentials with respect to the free curve by increasing the concentration of the expired drugs. This gives an indication that the VB1 and VB2 act as a mixed inhibitor.

Corrosion parameters such as anodic (*β*_a_), cathodic (*β*_c_) Tafel slopes, corrosion potential (*E*_corr._), corrosion current density (*I*_corr._) and inhibition efficacy (% *P*_GP_) were determined from the GP curves and were recorded in Table 2. It is illustrated in this table, as the concentration of expired VB1 and VB2 increased, the values of *E*_corr._ are slightly diverted to a more negative direction. The displacement in *E*_corr._ (Δ*E*_corr._) between the blank acidic solution and contained the two expired dugs less than 85 mV. Therefore, the expired drugs examined were classified as mixed inhibitors.^40^ The values *β*_a_ and *β*_c_ are changed to the positive and negative potential but the cathode are more polarized because the values of *β*_c_ are greater than *β*_a_. This confirms that the expired VB1 and VB2 worked as mixed inhibitor mainly cathodic. The values of *I*_corr._ were lowered and the % *P* increased, giving it the inhibitory power of the examined expired drugs. The values of % *P* of VB2 were greater than that of VB1 in all concentrations examined and this explained in the inhibition interpretation part.

**Table 2:** GP data for SABIC iron corrosion in free 0.5 M H_2_SO_4_ solution and with some concentrations of the expired VB1 and VB2

Inhibitors	Drug conc. (mg l^−1^)	*β*_a_ (mV per decade)	−*β*_c_ (mV per decade)	−*E*_corr._ (mV (SCE))	*I*_corr._ (μA cm^−2^)	% *P*_GP_
—	0	110	118	485	632	—
VB1	50	115	126	490	238	62.34
	100	120	138	492	196	68.98
	150	132	146	496	142	77.53
	200	140	162	502	78	87.66
	250	155	175	506	56	91.14
VB2	50	118	134	495	218	65.51
	100	123	146	502	162	74.36
	150	138	155	506	97	84.65
	200	150	170	508	66	89.55
	250	162	185	512	48	92.40

### 3.2. PDAP measurements

**Fig. 2:**
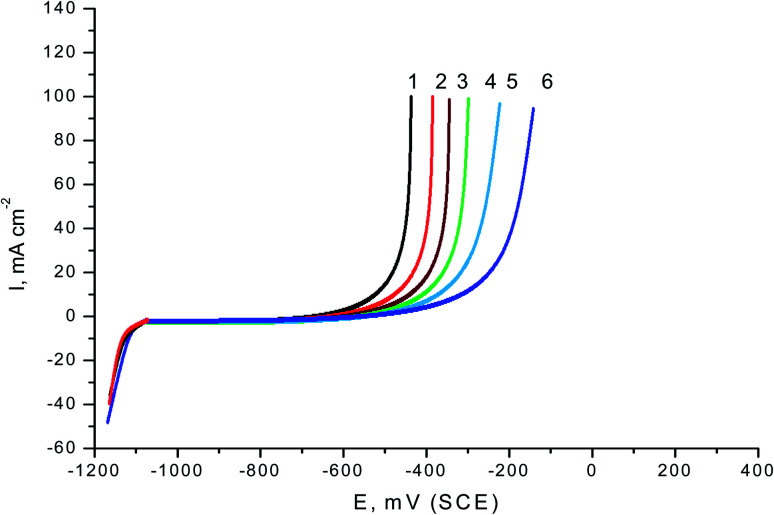
PDAP curves of SABIC iron in 0.5 M H_2_SO_4_ including 0.5 M NaCl solutions including various concentrations of VB2 at scan rate 1 mV s. (1) 0.00 mg l^−1^, (2) 50 mg l^−1^, (3) 100 mg l^−1^, (4) 150 mg l^−1^, (5) 200 mg l^−1^ and (6) 250 mg l^−1^.

The two expired drugs (VB1 and VB2) were tested as pitting inhibitor for the localized attack of SABIC iron in the 0.5 M H_2_SO_4_ including 0.5 M NaCl as pitting corrosion agent. Fig. 2 displays the PDAP curves for SABIC iron in 0.5 M H_2_SO_4_ + 0.5 M NaCl solution in the devoid of and the existence of some concentration ranging from 50 to 250 mg l^−1^ of expired VB1 and VB2. The scanning rate is adjusted at 1 mV sec^−1^. Analogous curves were obtained for the other expired VB1 but not visible. The general features of this figure, there is absence of any dissolution peak during anodic scan. The current remains constant due to the stability of the film formed on the surface of the SABIC iron until this film is destroyed at certain potential and the current is increased to more positive values. This potential is called, the pitting potential (*E*_pit._).^41^ This confirms that a pitting attack has occurred.

**Fig. 3:**
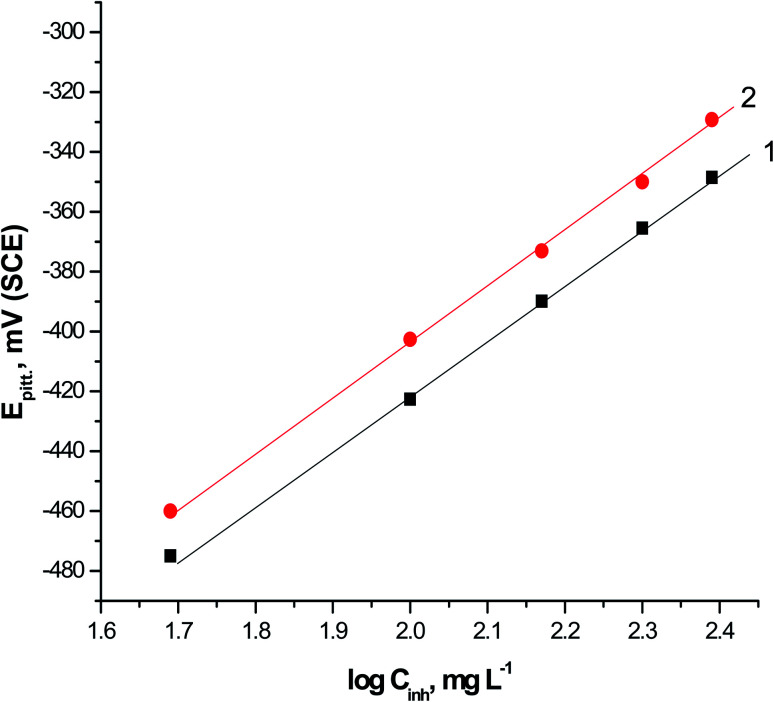
The relationship between *E*_pit._ and log *C*_inh_ for SABIC iron in 0.5 M H_2_SO_4_ + 0.5 M NaCl solutions containing various concentrations of VB1 and VB2.

The correlation between the *E*_pit._ and the logarithmic concentrations of expired VB1 and VB2 is displayed in Fig. 3. The straight lines relationship was obtained in case of the two expired vitamins according to the subsequent equation.^41,42^

8 *E*_pit._ = *α* + *δ* log *C*_drug_

where, *α* and *δ* are constants relying on the nature of the electrode and the type of additives applied. From Fig. 3 it is obvious that, more positive shift in *E*_pit._ as the concentrations of the expired VB1 and VB2 increase. This confirm that the pitting attack caused by Cl^−^ ions is inhibited by the existence of two expired vitamins. A more positive shift in *E*_pit._ in the presence of VB2 than VB1. This means that the VB2 is inhibited the pitting corrosion of SABIC iron more than VB1.

### 3.3. EIS measurement

**Fig. 4:**
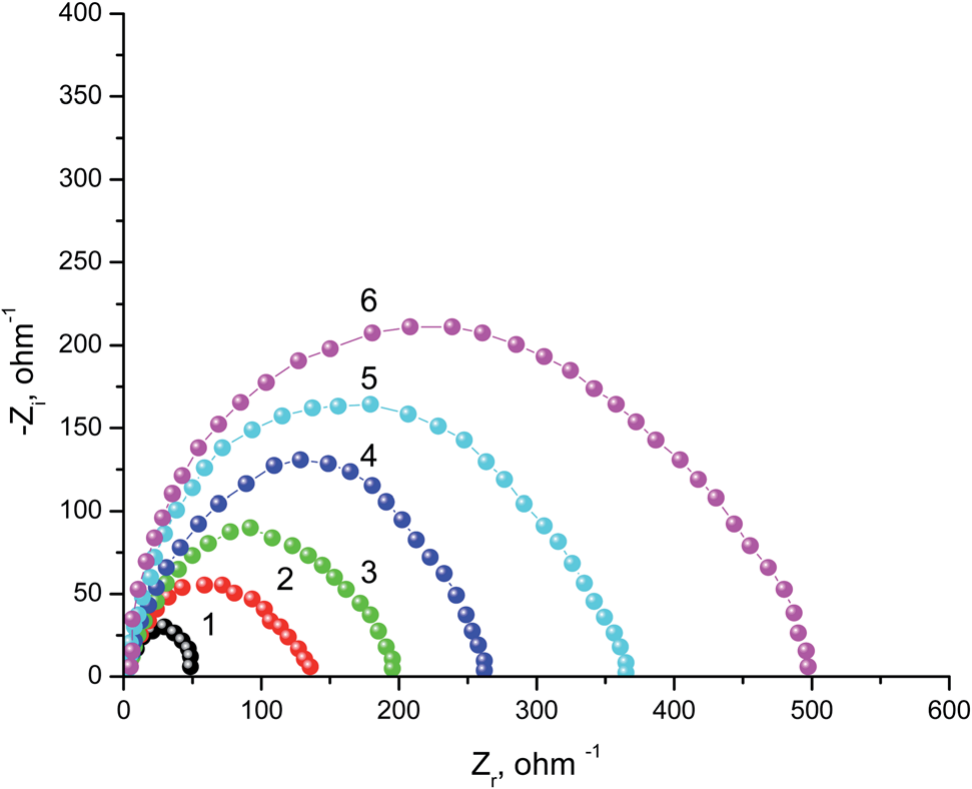
Nyquist plots SABIC iron corrosion in the blank 0.5 M H_2_SO_4_ and with addition of certain concentrations of VB2. (1) 0.00 mg l^−1^, (2) 50 mg l^−1^, (3) 100 mg l^−1^, (4) 150 mg l^−1^, (5) 200 mg l^−1^ and (6) 250 mg l^−1^.

The behavior of SABIC iron in a solution of 0.5 M H_2_SO_4_ without and with some concentrations of VB2 was shown through as a single depressed capacitive semicircle in the EIS diagrams (Fig. 4). Similar figure was acquired for the other expired VB1 but not visible. The resulted semicircles seem to be imperfect due to the heterogeneity, the frequency dispersion and the roughness of the studied samples.^43^ Also, it was shown from the Nyquist plots that the iron corrosion reaction is under charge transfer mechanism in both inhibited and un inhibited solutions due to the similarities of resulted.^44,45^

The equivalent circuit that fit the given plots is Randles (*R*_s_(*R*_ct_/CPE)) with a single time constant. Whereas,the solution resistance *R*_s_, the charge transfer resistance *R*_ct_, CPE represents a constant phase element and the double layer capacitance (*C*_dl_) are the semicircle parameters like these discussed before in literatures.^46–50^ The CPE is introduced to simulate the non-ideal capacitive behavior of SABIC iron/HCl interface, and its impedance is defined as follows:^49^9
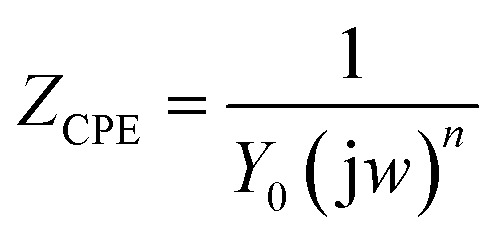
where *Y*_0_ is a CPE constant, *j* is the imaginary number, *ω* is the angular frequency (*ω* = 2π*f*, *f* represents the AC frequency in Hz), *n* is a phase shift, which is concerned to the system homogeneity, when the CPE display a pure capacitance, *n* = 1 and the *C*_dl_ was determined by the following equation:^50^10
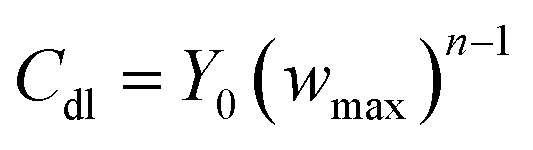


All the parameters obtained from EIS plots were given in Table 3, which showed a good consistence with that obtained from the GP method. The increase in *R*_ct_ from which the IE% was calculated and also, the decrease in *C*_dl_ proves the ability of the studied vitamins to reduce the aggressive action of the acid medium.

**Table 3:** EIS parameters for SABIC iron corrosion in free 0.5 M H_2_SO_4_ solution and with certain concentrations of the expired VB1 and VB2

Inhibitors	Drug conc. (mg l^−1^)	*R*_ct_ (Ω cm^2^)	*C*_dl_ × 10^−3^ (μF cm^−2^)	% *P*_EIS_
0.5 M HCl	0.0 mg l^−1^	46	433	—
VB1	50 mg l^−1^	123	97	62.60
	100 mg l^−1^	168	84	72.61
	150 mg l^−1^	211	68	78.19
	200 mg l^−1^	308	58	85.06
	250 mg l^−1^	423	42	89.12
VB2	50 mg l^−1^	143	91	67.83
	100 mg l^−1^	198	78	76.76
	150 mg l^−1^	263	61	82.50
	200 mg l^−1^	366	49	87.43
	250 mg l^−1^	503	41	90.85

Also, it was shown from the values given in Table 3, that the optimum concentration of the studied inhibitor that a chive the highest % *P* is the one with highest value of *R*_ct_ and lowest of *C*_dl_; and hence, the effectiveness of vitamin adsorption on the surface of the SABIC iron which leading to suppress the corrosion process.

According to the Helmholtz model, the reduce of the *C*_dl_ value (eqn (11)) indicates an increase in the double layer thickness (*d*), which can be referred to the development of a compact protective film on the metal surface by the inhibitor adsorption.^51^11
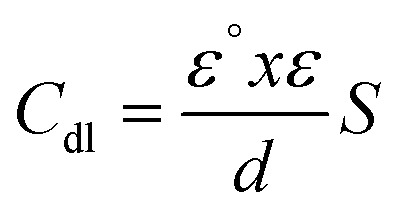
where *d* is the thickness of the film, *ε*° is the permittivity of the air, *ε* is the medium dielectric constant, and *S* represents the surface area of the working electrode.

**Fig. 5:**
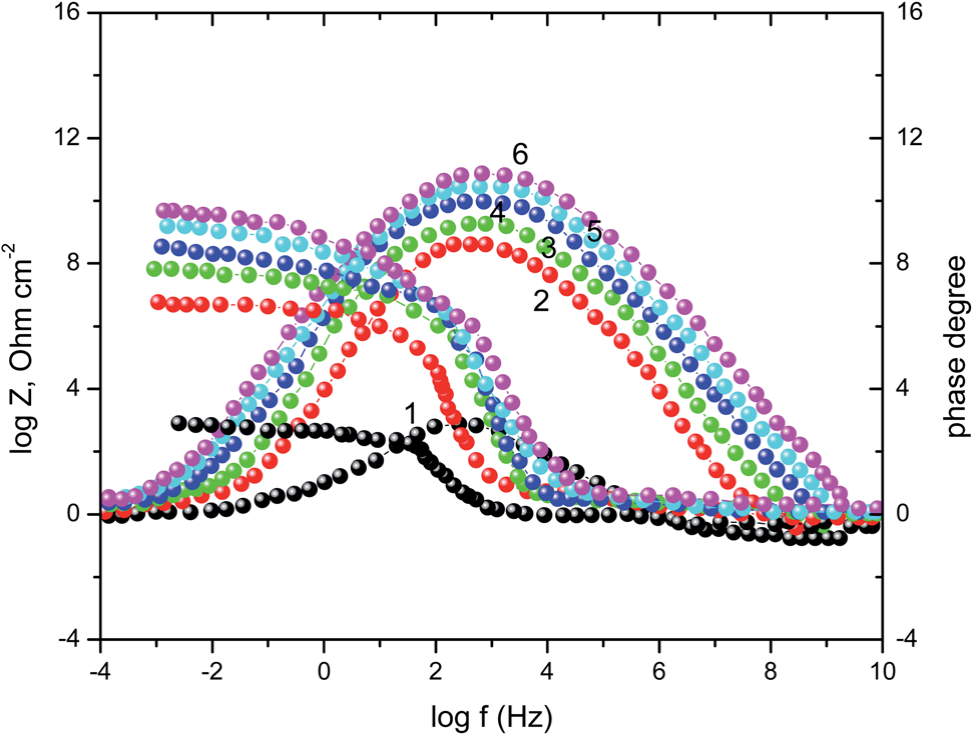
Nyquist plots SABIC iron corrosion in the blank 0.5 M H_2_SO_4_ and with addition of certain concentrations of VB2. (1) 0.00 mg l^−1^, (2) 50 mg l^−1^, (3) 100 mg l^−1^, (4) 150 mg l^−1^, (5) 200 mg l^−1^ and (6) 250 mg l^−1^.

A complementary information's that can cover the discussion of the corrosion process in the view of EIS technique were given from Bode plots Fig. 5. The shows an increase of the impedance modulus, at low frequencies, with the inhibitor concentration that improving the adsorption of the vitamin molecules on the surface of SABIC iron and hence, block the active sites against the corrosive medium.^52^ In addition, there is only one peak in the phase angle plots that elucidated the presence of a single time constant at the metal/solution interface.

### 3.4. WR measurements

#### 3.4.1. Impact of concentration of the expired vitamins

**Fig. 6:**
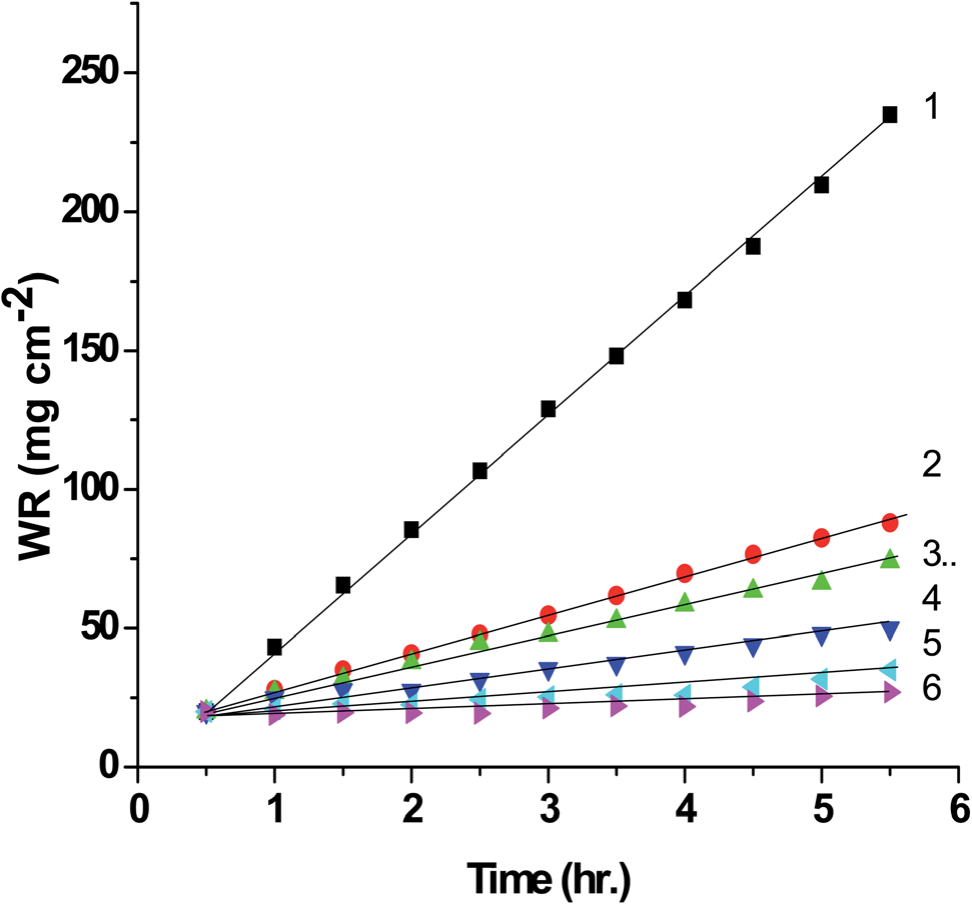
WR–time curves of SABIC iron in 0.5 M H_2_SO_4_ solution containing some concentrations of VB2 at 303 K. (1) 0.00 mg l^−1^, (2) 50 mg l^−1^, (3) 100 mg l^−1^, (4) 150 mg l^−1^, (5) 200 mg l^−1^ and (6) 250 mg l^−1^.

The correlation between WR and time for SABIC iron in the free 0.5 M H_2_SO_4_ solution and includes some concentrations ranging from 50 ppm to 250 ppm of VB2 was displayed in the Fig. 6. Analogous curves were obtained for the other expired VB1 but not visible. A linear relationship is obtained which indicates that there is no insoluble surface layer during corrosion.

The general observation of this Fig. 6 the WR decreased with increasing the concentration of the expired vitamins. Also, the values of % *P* and *θ* as determined from eqn (2) and (3) increases. This confirm the inhibitory impact of expired VB1 and VB2. This can be demonstrated by the adsorption of the expired vitamins on SABIC iron surface. Hence, the surface of SABIC iron is separated from the corrosive electrolyte by creating a film on its surface.^53,54^ The % *P* values of VB2 is more than VB1. These results are in agreement with those obtained from GP, EIS and PDAP measurements. The % *P* values of VB1 and VB2 is more inhibition efficacy than expired omeprazole for corrosion of SABIC iron in acidic.^28^ The expired ampicillin and ceftriaxone drugs^26^ are more inhibition efficacy than the investigated expired vitamins. The high % *P* is due to the presence of several active center for adsorption in the chemical structure of the expired drugs.

#### 3.4.2. Impact of temperature

The impact of elevated temperature ranging from 303 to 318 K on the corrosion rate determined from WR of SABIC iron in 0.5 M H_2_SO_4_ solution comprising 250 mg l^−1^ of VB1 and VB2 using WR technology. Figures are identical to Fig. 6 were obtained but not visible. The values of *R*_corr._ and % *P* at elevated temperatures are recorded in Table 3. It is evident that, with increasing temperature the values of *R*_corr._ increases and % IE reduced. This demonstrated that the elevated temperature led to a decrease in the adsorption of the expired vitamins and thus accelerate of the corrosion process.^55^

The reduce in the % *P* as the temperature elevated confirms that the adsorption of two expired vitamins examined on the surface of SABIC iron is physical. This type of adsorption can be described as an electrostatic interaction between the expired vitamins and charged sites on the SABIC iron surface. The increase in temperature results in desorption of some adsorbed expired VB1 and VB2 molecules, thus the covered area of the metal decreases resulting in a reduce the % *P* (Table 4).

**Table 4:** Corrosion parameters acquired from the corrosion of SABIC iron in 0.5 M H_2_SO_4_ solution including different concentrations of VB1 and VB2 using WR technique at 303 K

Inhibitors	Inhibitors conc. (mg l^−1^)	*R*_corr._ (mg cm^−2^ min^−1^)	*θ*	% *P*_WR_
H_2_SO_4_	0.5 mg l^−1^	0.294	—	—
VB1	50 mg l^−1^	0.119	0.595	59.52
	100 mg l^−1^	0.098	0.666	66.66
	150 mg l^−1^	0.068	0.769	76.87
	200 mg l^−1^	0.041	0.861	86.05
	250 mg l^−1^	0.031	0.895	89.45
VB2	50 mg l^−1^	0.110	0.626	62.58
	100 mg l^−1^	0.087	0.704	70.41
	150 mg l^−1^	0.056	0.809	80.95
	200 mg l^−1^	0.033	0.888	88.77
	250 mg l^−1^	0.025	0.915	91.49

The activation energy (*E*_a_) for the corrosion of SABIC iron in 0.5 M H_2_SO_4_ solution containing 250 mg l^−1^ of VB1 and VB2 using WR technology were calculated using Arrhenius equation.^56,57^12
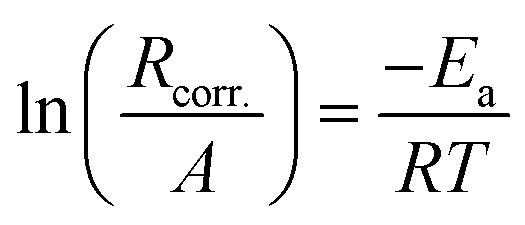
where, *R*_corr._ represents the rate of corrosion reaction, *A* is the Arrhenius constant, *R* is the gas constant and *T* is the absolute temperature.

**Fig. 7:**
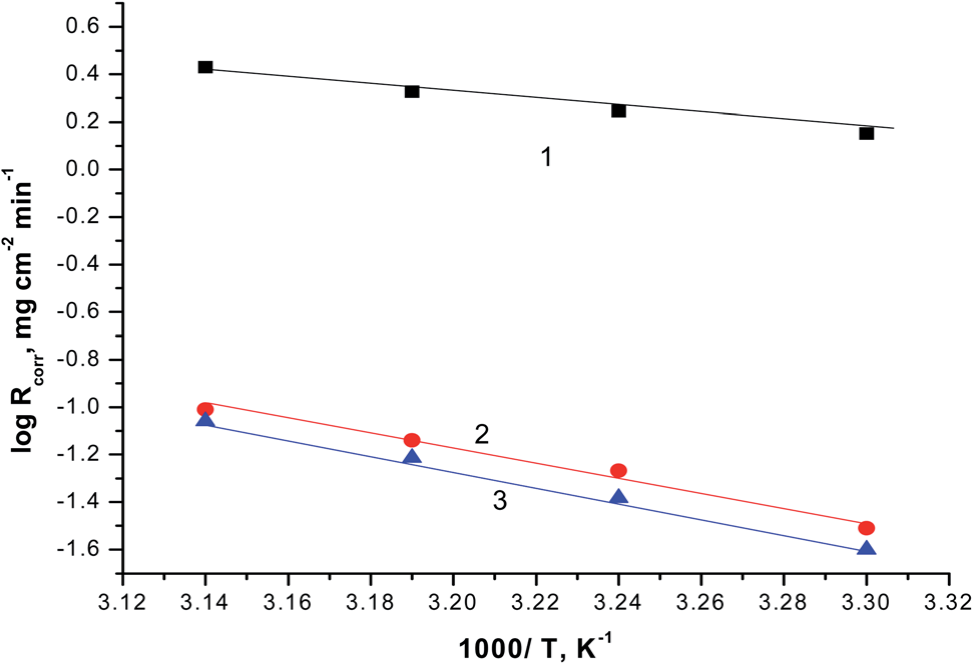
The correlation between the *R*_corr._ and 1/*T* of SABIC iron in 0.5 M H_2_SO_4_ solution devoid of and containing 250 mg l^−1^ of VB1 and VB2. (1) 0.5 M H_2_SO_4_, (2) VB1 and (3) VB2.

The relationship between the *R*_corr._ and 1/*T* are represented graphically in Fig. 7. Straight lines were acquired with slope equal (−*E*_a_/*R*). The values of *E*_a_ is equal to 29.45 kJ mol^−1^ in case of 0.5 M H_2_SO_4_ solution where in the existence of expired vitamins VB1 and VB2 are equal to 35.46 and 38.34 kJ mol^−1^, respectively. The increase of *E*_a_ in the existence of expired vitamins relative to the blank solution due to the construction of a thickly adsorbed layer of expired vitamins on the surface of SABIC iron. These data demonstrate that the expired VB1 and VB2 act as inhibitors by increasing activation energy of dissolution of SABIC iron by creating a barrier to mass and charge transfer by adsorbing them on the surface of SABIC iron (Table 5).

**Table 5:** Impact of elevated temperature on the *R*_corr._ and % *P* obtained from the corrosion of SABIC Fe in 0.5 M H_2_SO_4_ solution containing 250 mg l^−1^ of VB1 and VB2 using WR technique

Medium	*T* (K)	*R*_corr._ (mg cm^−2^ min^−1^)	% *P*_WR_
0.5 M H_2_SO_4_	303	0.294	—
	308	0.318	—
	313	0.350	—
	318	0.368	—
0.5 M H_2_SO_4_ + 250 mg l^−1^ of VB1	303	0.031	89.45
	308	0.056	82.39
	313	0.072	79.43
	318	0.097	73.64
0.5 M H_2_SO_4_ + 250 mg l^−1^ of VB2	303	0.025	91.49
	308	0.047	86.57
	313	0.069	80.28
	318	0.088	76.08

The variation in the values of standard activation enthalpy (Δ*H*°) and standard activation entropy, (Δ*S*°) were estimated using the next transition state equation.^56,57^13
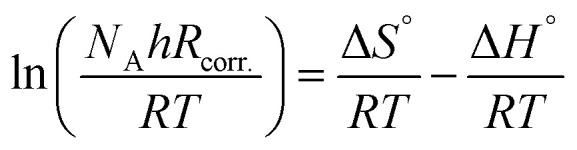
where, *N*_A_ and *h* are the Avogadro's number and the Plank constant.

**Fig. 8:**
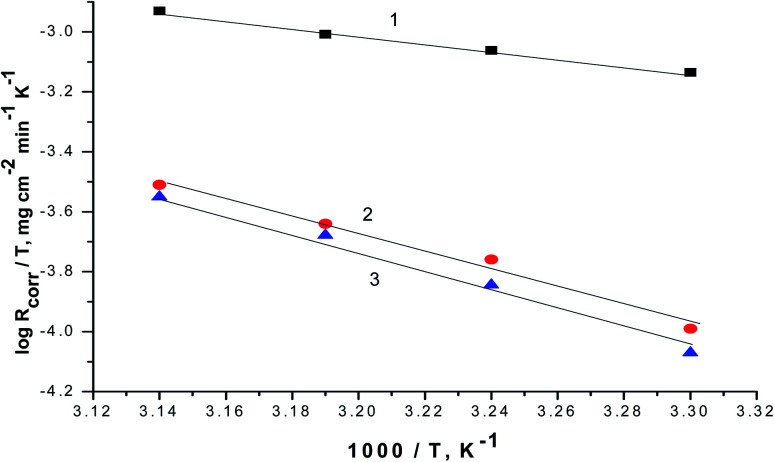
The correlation between the *R*_corr._/*T* and 1/*T* of SABIC iron in 0.5 M H_2_SO_4_ solution devoid of and containing 250 mg l^−1^ of VB1 and VB2. (1) 0.5 M H_2_SO_4_, (2) VB1 and (3) VB2.

Plotting of log(*R*_corr._/*T*) *versus* (1/*T*), gave straight lines as presented in Fig. 8 of the values of Δ*H*° are estimated from the slope of the straight lines and equal to 27.66 kJ mol^−1^ in case of 0.5 M H_2_SO_4_ solution where in the existence of expired vitamins VB1 and VB2 are equal to 32.67 and 36.42 kJ mol^−1^, respectively. The positive values of Δ*H*° reflect the endothermic nature of the activated complex formation during the corrosion process.

The Δ*S*° are estimated from the intercept of the straight lines in Fig. 8 and equal to −0.288 J mol^−1^ K^−1^ in case of 0.5 M H_2_SO_4_ solution where in the existence of expired vitamins VB1 and VB2 are equal to −0.317 and 0.378 J mol^−1^ K^−1^ in, respectively. The negative sign of Δ*S*° elucidates that the activated compound in the rate determination step represents correlation, not disengagement. This demonstrates that the activated molecules were less randomness state than that at the initial stage.^58,59^

### 3.5. Adsorption isotherm

**Fig. 9:**
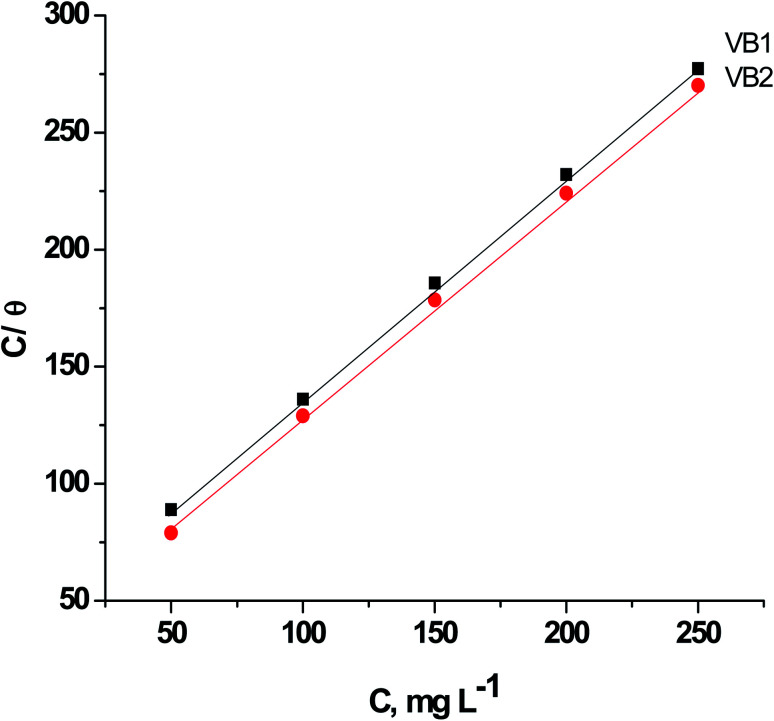
Relation between CVB/*θ versus* CVB for of SABIC iron in blank 0.5 M H_2_SO_4_ solution and including some concentrations of VB1 and VB2.

The adsorption of expired VB1 and VB2 on the SABIC iron surface is supposed as a substitute adsorption operation between the expired vitamins in the aqueous solution [VB_(aq)_] and the water molecules adsorbed on the electrode surface [H_2_O_(ads)_] owing to the subsequent equation:

14 VB_(aq)_ + *n*H_2_O_(ads)_ = VB_(ads)_ + *n*H_2_O_(aq)_

where, *n* is the proportion of number of water molecules replaced by single molecule of vitamins. To choose the preferable isotherm for adsorption of expired VB1 and VB2 on the SABIC iron. We insert the value of surface coverage (*θ*) in various isotherms such as Langumir, Frumkin, Frundlich and Temkin isotherms. We define that the Langmuir isotherm is the appropriate isotherm due to the following equation:

15 *C*_VB_/*θ* = *C*_VB_ + 1/*K*_ads_

where, *C*_VB_ is the concentration of the tested expired vitamins, *K*_ads_ is the equilibrium constant of adsorption. Fig. 9 explicate the correlation between *C*_VB_/*θ versus C*_VB_. This relation gives straight lines with slopes equal unity signalizing that the adsorption of on VB1 and VB2 on the SABIC iron in 0.5 MH_2_SO_4_ solution obeys Langmuir isotherm.

This isotherm postulates monolayer adsorption hence zero interaction between the adsorbate species on the SABIC iron surface. Moreover, the value of *K*_ads_ can be determined from the intercept and equal to 0.0385 and 0.0625 for VB1 and VB2, respectively. The standard free energy of adsorption 
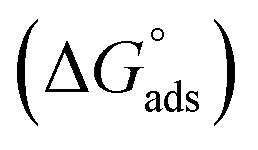
 was determined by the subsequent equation:16
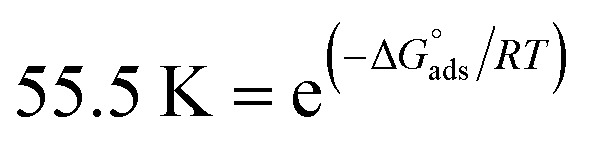
where: *R* is the universal gas constant, *T* is the absolute temperature. The value 55.5 is the concentration of water in mol l^−1^. The computed values of 
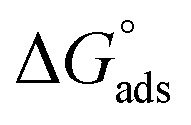
 are equal to −21 502 and −31.36 kJ mol^−1^ for VB1 and VB2, respectively. The acquired negative values of 
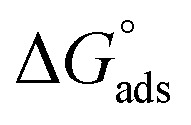
 confirm that the adsorption of VB1 and VB2 on the SABIC iron are spontaneous.

### 3.6. Interpretation of inhibition

The inhibition strength of the two expired VB1 and VB2 towards the corrosion of SABIC iron in 0.5 M H_2_SO_4_ solution is mainly interpreted by its adsorption on the iron surface. Chemical and electrochemical technologies were applied to calculate the % *P*. The % *P* increases as the concentration of expired vitamins increases due to the area covered by the adsorbed molecules increases. The adsorption can be proceed by a construction of adsorbed layer between the SABIC iron surface and aggressive H_2_SO_4_ solution. This layer prevents the mass and charge transformation between the SABIC iron and its surrounding. The inhibitory efficacy reduces with increasing temperature. Moreover, the activation energy increases upon addition of the expired vitamins. Such results indicate that the adsorption of expired vitamins on the metal surfaces is physical in nature. The adsorption of expired vitamins depends on some factors like the chemical composition of the vitamin and the occurrence of some active centers in its structure, and ability to form complexes.^41^

**Scheme 1:**
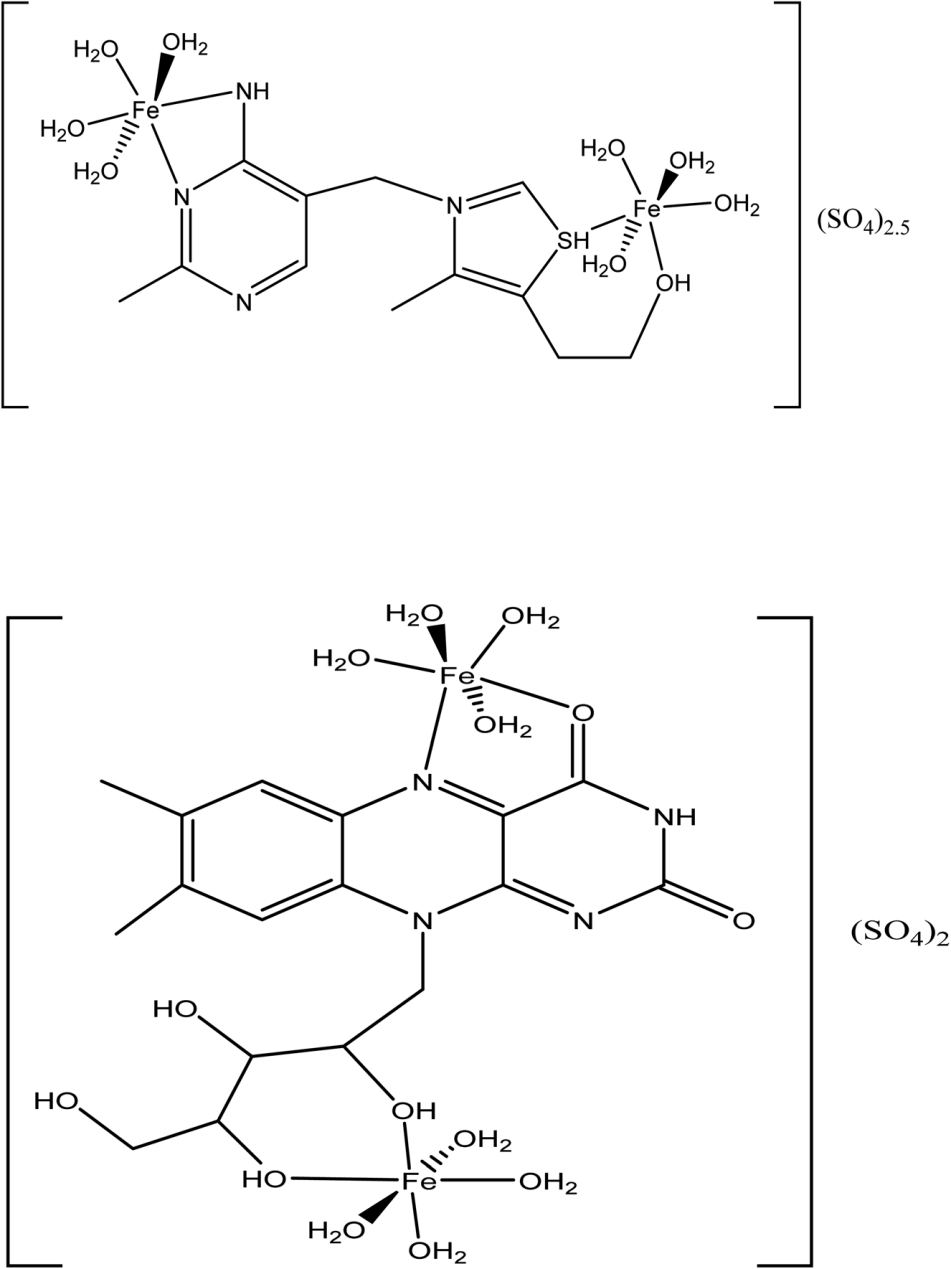
Proposed structures for the complexes formed between the SABIC iron and (A) VB1 and (B) VB2.

It is worth mentioning that stability of complexes having chelate rings is much more than that of complexes containing none or fewer chelate rings owing to the chelate effect.^60,61^ Therefore, one can expect the following for expired VB1: there are two possible coordination sites for iron as shown in Scheme 1A. For the first coordination site, iron will prefer to coordinate to the two nitrogen atoms of the amino and imine groups of the VB1 due to the chelate effect; the coordinated iron atom will complete its coordination sphere with 4H_2_O molecules since this is occurring in the aqueous media. While for the second coordination site for this compound iron can coordinate to the –S and –OH function groups of the thiamine compound forming six-membered chelate ring. Iron can then complete its coordination sphere similarly with 4H_2_O molecules. However, the latter chelate is more stable than the former one since the six-membered ring is much more stable than the four-membered ring in chelation. On the other hand, expired VB2 can also form a dinuclear complex with iron metal through two possible coordination sites, as shown in Scheme 1B. Iron can coordinate to –C=O and imine groups forming a five-membered chelate ring. The other coordination site, iron can coordinate to two –OH groups forming a six-membered chelate ring. Owing to the chelate effect, the second site is much more stable than the first site. Each iron atom has a coordination number of six and consequently both iron atoms can complete their coordination sites by H_2_O molecules. The oxidation states of the coordinated iron are +2. Moreover, both iron-VB1 and iron-VB2 chelates charges could be neutralized by sulfate anions as counter ions since the media for both reactions were sulfuric acid aqueous solutions. The proposed structures of complexes formed between SABIC iron and expired VB1 and VB2 forces the expired vitamins to be adsorbed onto the surface of SABIC iron which leads to increase in the inhibition effectiveness by increasing the iron surface coverage with an inhibitor.

### 3.7. DFT study

**Fig. 10:**
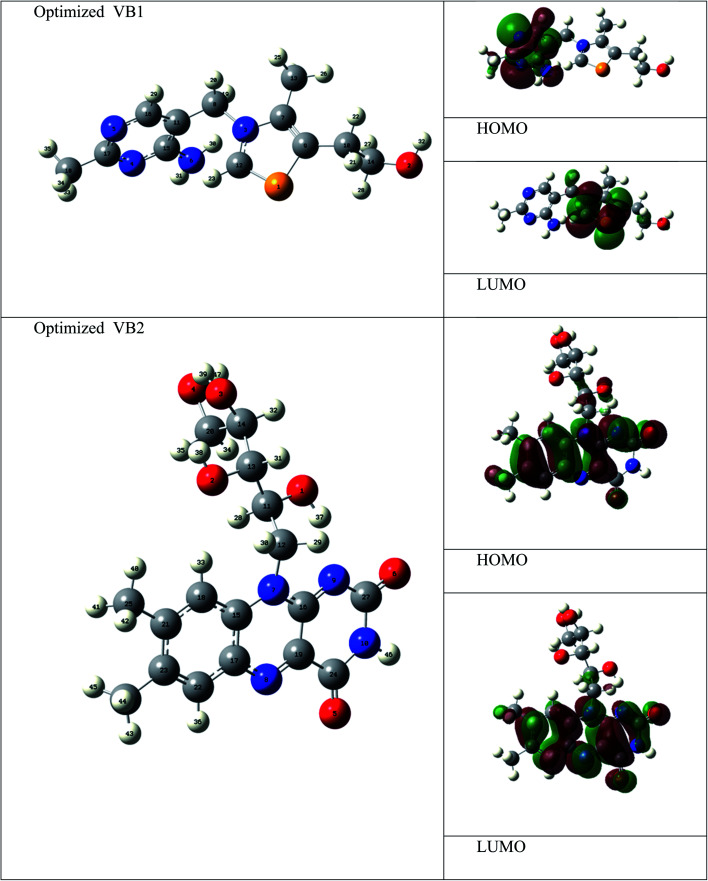
Optimized geometry, HOMO and LUMO Frontier orbitals of VB1 and VB2 inhibitors.

The electronic and geometrical structures of inhibitor molecules influence its inhibitory performance. DFT study was employed to study the electronic properties of VB1 and VB2 molecules. The HOMO and LUMO plots for the VB1 and VB2 molecules were shown in Fig. 10. The energies of HOMO and LUMO parameters were used to predict the chemical reactivity of the inhibitor. The HOMO energy gives a sign of donating an electron to the empty d-orbital of the metal surface, while LUMO energy of accepting an electron from the metal surface.^62,63^ Therefore, the higher HOMO and the lower LUMO energies indicate a higher inhibition efficiency. As collected in Table 6, the VB2 molecule has higher HOMO and lower LUMO energies than VB1 in gas and aqueous solvent. So, VB2 molecule gives a higher inhibition efficiency than VB1 molecule.

**Table 6:** Quantum parameters of VB1 and VB2 inhibitors in gas and aqueous phase

	Gas phase		Aqueous phase	
	VB1	VB2	VB1	VB2
*E*_HOMO_ (eV)	−9.70	−6.44	−6.76	−6.40
*E*_LUMO_ (eV)	−5.76	−2.98	−2.20	−3.00
Δ*E* (eV)	3.94	3.46	4.56	3.40
μ (Debye)	5.51	6.96	7.44	10.21
*η* (eV)	1.97	1.73	2.28	1.70
*σ* (eV^−1^)	0.51	0.58	0.43	0.59
Δ*N* (eV)	−0.74	0.03	0.07	0.03

According to Frontier molecular orbital, the HOMO and LUMO which affects the adsorption behavior of a molecule through interaction with conduction and valence band of the metal surface, respectively. As was seen in Fig. 10, the electron density distribution in the HOMO and LUMO of VB2 is mostly distributed on alloxazine regions indicating electron transfer from HOMO to LUMO with bonding formation between the studied reagents.^64^ For VB1 molecule, HOMO is localized on the pyrimidine ring and LUMO is located on the thiazole ring. Therefore, electron donation capability of VB2 molecule is greater than VB1 molecule, this leads to VB2 molecule exhibit greater interaction with SABIC iron than VB1 molecule.

The energy gap is one of the important quantum descriptors which correlate with the inhibition efficacy of the compound. The molecule that has a high value in the energy gap indicates the stability of the molecule and the low value in the energy gap indicates that the compound has a high efficacy. As shown in Table 6, the VB2 is lower in the energy gap than the VB1 molecule in a gaseous and aqueous medium. Therefore, VB2 inhibitor gives a higher efficacy than VB1 molecule. Also, VB2 has greater adsorption on the iron surface.^65^

The dipole moment of the compound is used to determine the direction of the inhibition process and has a relationship to the electron distribution in the molecule.^66^ The polar compounds that have a high dipole moment lead to high inhibition efficacy. As collected in Table 6, the comparison between the values of the dipole moment of both inhibitors VB1 and VB2 is found that the dipole moment of VB2 is greater than VB1. The dipole moment in the aqueous medium is greater than in the gaseous medium of both compounds.

The global hardness and softness are among the important quantum parameters that have a relationship with the reactivity and the stability of a compound. According to Lewis's theory, the molecule that has a low energy gap is a soft molecule and *vice versa* for the hard molecule.^67^ As present in Table 1, the VB2 inhibitor has higher softness and lower hardness than VB1 and this confirms that VB2 molecule give higher inhibition efficiency than VB1 which consistent with experimental results.

The fraction of electron transferred (Δ*N*) is the other important parameter that results when the inhibitor molecule is close to the metal surface. The fraction of electron transferred for expired VB1 and VB2 were estimated in the gas and aqueous medium. As seen in Table 6, the positive and negative values of Δ*N* indicates that the inhibitors act as electron donor and acceptor. The electron donation of inhibitor increases if Δ*N* < 3.6.^68^ As shown in Table 1, the VB2 has the capability of electron donation in the gas phase but VB1 act as the electron acceptor. In the aqueous phase, VB1 and VB2 inhibitors act as electron donor.

Molecular electrostatic potential (MEP) and Fukui function were effective tools to predict the reactive centers for nucleophilic and electrophilic behavior. As given in Table 7, for VB2 molecule the maximum value of *f*^−^ and *f*^+^ is C12 atom which responsible for both electrophilic and nucleophilic attack. For VB1, the maximum *f*^+^ value on C8 and C11 atom and the maximum *f*^−^ on C14 and C18 atoms.

**Table 7:** Fukui indices of VB1 and VB2 inhibitors

	VB1	*f* ^+^	*f* ^−^		VB2	*f* ^+^	*f* ^−^
1	S	−0.26168	−0.06608	1	O	−0.0209	−0.0289
2	O	−0.02478	−0.09339	2	O	0.010475	0.004461
3	N	−0.0274	0.008998	3	O	−0.00604	−0.02415
4	N	−0.02162	−0.05833	4	O	−0.00943	−0.01583
5	N	−0.02702	−0.09706	5	O	−0.07157	−0.05406
6	N	0.018454	−0.06299	6	O	−0.07189	−0.08731
7	C	−0.01274	−0.01015	7	N	−0.01515	−0.02398
8	C	0.039473	−0.00239	8	N	−0.08922	−0.02243
9	C	−0.01716	−0.00321	9	N	−0.02862	−0.07788
10	C	0.023312	0.012522	10	N	0.006766	0.002449
11	C	0.039506	−0.02941	11	C	0.009059	0.013691
12	C	−0.14275	0.009736	12	C	0.022913	0.023635
13	C	0.003272	0.006362	13	C	0.00385	0.004964
14	C	−0.00385	0.021536	14	C	−0.00115	0.005587
15	C	−0.00316	−0.0358	15	C	−0.03888	−0.02603
16	C	−0.00656	−0.0363	16	C	−0.04745	−0.0205
17	C	−0.00249	−0.03649	17	C	0.010398	−0.02899
18	C	0.003644	0.012458	18	C	−0.02538	−0.02911
				19	C	−0.0453	−0.03496
				20	C	−0.00481	−0.00257
				21	C	−0.02048	−0.00834
				22	C	−0.03328	−0.01767
				23	C	0.000736	−0.02576
				24	C	−0.05581	−0.02749
				25	C	0.008285	0.006486
				26	C	0.005709	0.010209
				27	C	−0.03864	−0.03294

**Fig. 11:**
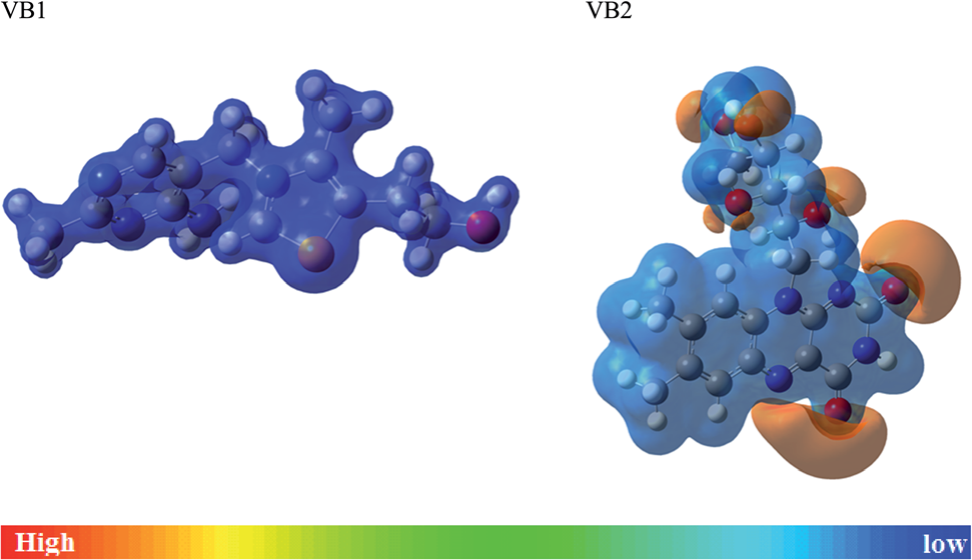
MEP maps of the VB1 and VB2 inhibitors.

MEP is another useful indicator for reactive centers of electrophilic and nucleophilic. As seen in Fig. 11, the blue region for positive electrostatic potential while the red region for negative electrostatic potential. The negative electrostatic is concentrated on oxygen atoms of VB2. The positive electrostatic potential is located in all regions for VB1 and VB2 molecules.

### 3.8. Protonated form

**Fig. 12:**
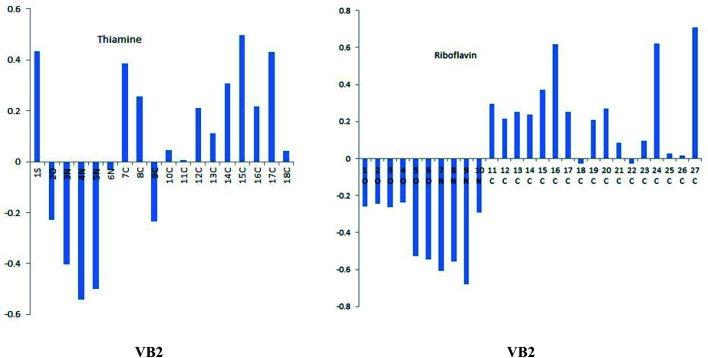
The Mulliken charges of the VB1 and VB2 inhibitors.

**Fig. 13:**
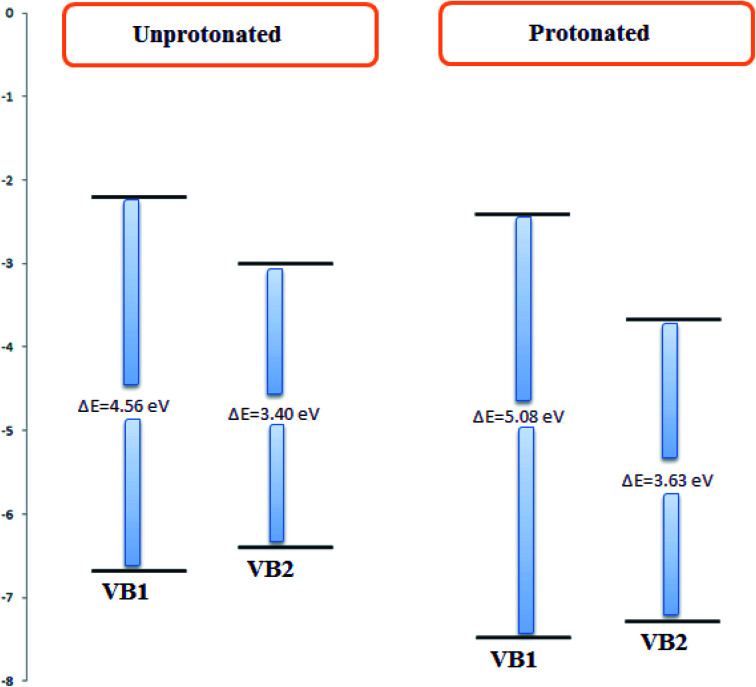
The unprotonated and protonated forms of VB1 and VB2 inhibitors.

VB1 and VB2 molecules contain heteroatom in its structure. These molecules in acidic solution undergo protonation. To know which atoms in a molecule will be protonated, the Mulliken atomic charges on N9 for VB2 and N4 of VB1 inhibitors as seen in Fig. 12, have a highly negative charge therefore, it is easily protonated through these atoms in an acidic solution. The quantum parameters derived from the protonated form have been shown in Fig. 13, the HOMO and LUMO values of the protonated were compared with the neutral forms. The HOMO values of both inhibitors in protonated form decrease indicating the lowering tendency of the two inhibitors to donate, while the LUMO values decrease suggested its tendency of accepting electron increases. Also, the energy gap increased for protonated form.

### 3.9. MC simulations

**Fig. 14:**
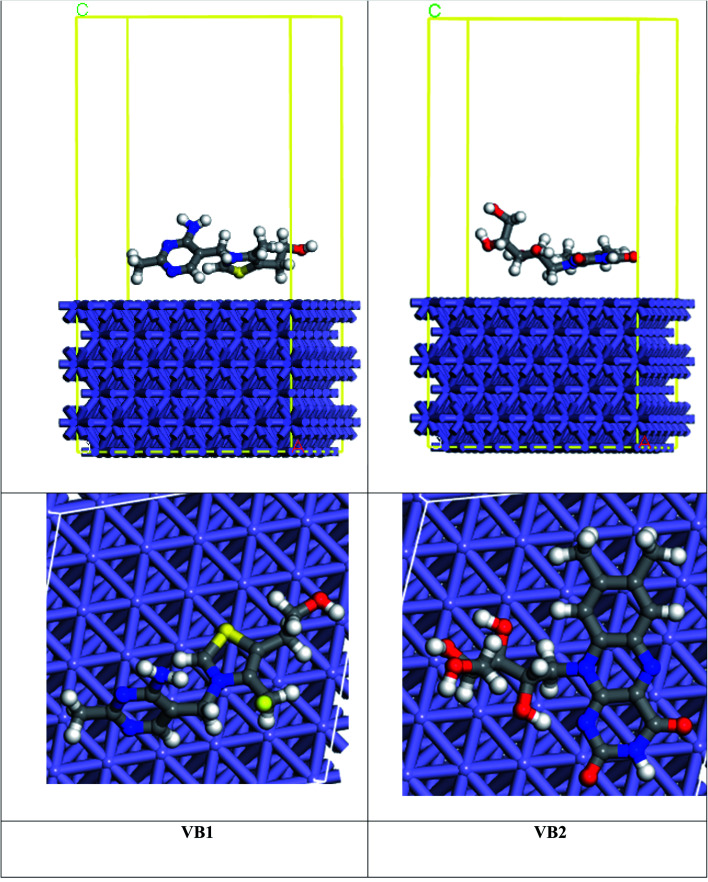
Side and top views of the interaction of VB1 and VB2 with Fe(110) surface.

MC simulation is a popular technique used to study the adsorption of VB1 and VB2 molecules on the SABIC iron surface. As shown in Fig. 14, the adsorption of VB2 is parallel though the alloxazine ring, and for VB1 the perpendicular adsorption through the pyrimidine ring but parallel for the rest of the inhibitor. The energy resulted from the interaction of VB1 and VB2 with the Fe(110) surface was determined as follow:

17 *E*_ads_ = *E*_inh+Fe(110)_ − *E*_Fe(110)_ − *E*_inh_

where *E*_inh+Fe(110)_, *E*_Fe(110),_ and *E*_inh_ are the energies of inhibitor on iron surface, the energy of iron surface, and the energy of inhibitors respectively.

The binding energy of the inhibitors on the Fe(110) surfaces is given by:

18 *E*_binding_ = −*E*_ads_

The high interaction energy between the inhibitor molecule and Fe surface reflects good adsorption on the Fe surface.^69^ As seen in Table 8, the binding energy of VB2 with Fe(110) is higher than VB1 inhibitor which coincides with the experimental inhibition efficacy and this result is confirmed by bond distance between the inhibitor and the Fe surface. VB2 has the bond distance between Fe–S1 (2.00 Å) shorter than VB1 inhibitor that has bond distance between Fe–H29 (2.42 Å).

**Table 8:** The adsorption and binding energies of expired VB1 and VB2 on Fe(110) surface

	*E*_ads_ (kcal mol^−1^)	*E*_binding_ (kcal mol^−1^)
Fe(110) + VB1	−119.14	119.14
Fe(110) + VB2	−172.66	172.66

## 4. Conclusions

Expired VB1 and VB2 acted as an efficacious inhibitor for the SABIC iron corrosion in 0.5 M H_2_SO_4_ solution. The galvanostatic polarization showed that the expired vitamins act as inhibitors of the mixed type. The inhibitory power of the tested vitamins was demonstrated by their spontaneous adsorption onto the surface of SABIC iron. Expired VB1 and VB2 act as pitting inhibitors for corrosion of SABIC iron. The interaction between SABIC iron and the two examined vitamins was further examined by quantum chemical calculation for a better understanding. Comparing the practical results with theoretically calculated showed that the VB2 molecule gives higher inhibition efficiency than VB1 molecule through some quantum parameters. VB2 molecule on Fe(110) surface shows higher binding energy than VB1 molecule.

## Conflicts of interest

The authors declare that there is no conflicts of interests regarding the publication of this manuscript.

## Supplementary Material
